# Lactate Metabolism: Separating Correlation From Causation

**DOI:** 10.1002/bies.70181

**Published:** 2026-09-09

**Authors:** Bhavya Blaze, Ahmad A. Cluntun

**Affiliations:** ^1^ Department of Biochemistry and Molecular Biology Rutgers Robert Wood Johnson Medical School Rutgers The State University of New Jersey Piscataway New Jersey USA

**Keywords:** aerobic glycolysis, clinical biomarkers, correlation versus causation, lactate metabolism, metabolic flux, mitochondrial metabolism, redox homeostasis

## Abstract

Lactate is among the most frequently measured metabolites in physiology and medicine, yet it remains one of the most persistently misunderstood. Long regarded as a metabolic waste product, a marker of anaerobic metabolism, or a direct cause of acidosis and fatigue, lactate remains burdened by misconceptions that obscure its true biological roles. Recent advances in metabolic flux analysis, isotope tracing, hyperpolarized magnetic resonance spectroscopy, and cellular imaging have revised this view and established lactate as a central intermediary in energy metabolism, redox homeostasis, and interorgan metabolic communication. Here, we re‐examine common myths surrounding lactate biology and clarify the distinction between correlation and causation in its interpretation. By reframing lactate as a dynamic indicator and, in some contexts, a regulator, of metabolic state rather than a toxic by‐product, this review improves experimental reasoning and clinical interpretation across diverse physiological and pathological contexts.

## Graphical abstract

Elevated lactate is often interpreted as the cause of pathology, when it more commonly reflects underlying metabolic state. This review separates correlation from causation and highlights how advances in technology have transformed our understanding of lactate biology.

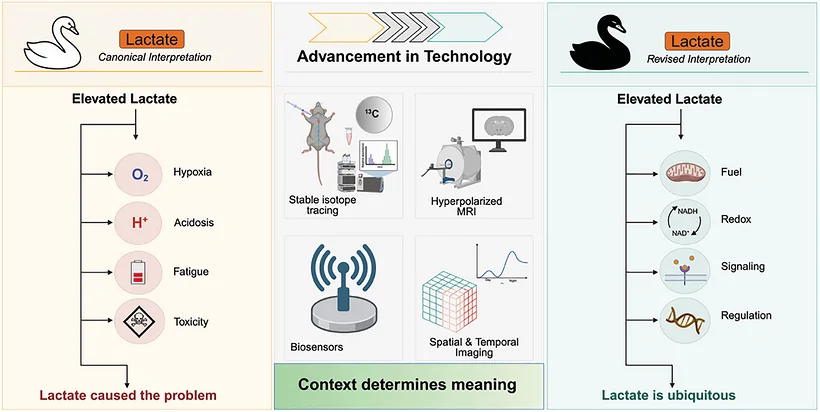

## 1 Introduction

Physiological homeostasis depends on the coordinated regulation of metabolism across multiple spatial and temporal scales, from intracellular reactions to whole‐organism energy balance. Disruption of this coordination underlies many pathological states, in which cells and tissues adopt compensatory metabolic responses to maintain function. Changes in metabolite concentrations often accompany these responses and can provide insight into underlying metabolic states. Among these metabolites, lactate is particularly misunderstood. Lactate is continuously produced by virtually all mammalian cells and circulates at low millimolar concentrations even under resting conditions [1, 2, 3]. Importantly, relatively stable circulating lactate concentrations can coexist with extremely high rates of lactate turnover, production, and utilization, underscoring that metabolite concentration alone does not capture metabolic flux. Consistent with this view, lactate serves as a physiological fuel during development, including in the fetal circulation and placenta. Together, these contexts illustrate that lactate production is not inherently pathological [4]. Elevated lactate is commonly equated with oxygen deficiency, mitochondrial dysfunction, or tissue injury, while its physiological roles as a fuel, redox buffer, and metabolic intermediary are often overlooked.

Many of these misinterpretations stem from a fundamental error: the conflation of correlation with causation. Lactate rises in conditions of stress, exercise, and illness, but this co‐occurrence is often misinterpreted to mean that lactate itself drives pathology. Unlike glucose, whose elevation is readily interpreted as dysregulation, or ketone bodies, which are widely accepted as adaptive fuels, lactate resists simple classification as either beneficial or harmful. Addressing persistent misconceptions surrounding lactate is essential to improve both experimental interpretation and clinical decision‐making. In this review, we revisit common myths about lactate metabolism and clarify what lactate does and does not represent in physiology and disease. Many of these misconceptions persist because they are reinforced by historical terminology, simplified experimental readouts, and clinical heuristics rather than direct mechanistic evidence. This review focuses on how lactate is interpreted rather than on cataloging its molecular mechanisms, which are covered elsewhere.

## 2 Common Myths About Lactate

### 2.1 Myth 1: Lactate is Lactic Acid (or Lactose)

#### 2.1.1 Truth: Lactate, Not Lactic Acid, Predominates in Mammalian Physiology

Confusion surrounding lactate begins with terminology. Historically, lactic acid was first isolated from sour milk, which embedded the Latin root *lac* into metabolic nomenclature. This linguistic legacy fostered the incorrect assumption that lactate, lactic acid, and lactose are interchangeable biological entities.

**FIGURE 1:**
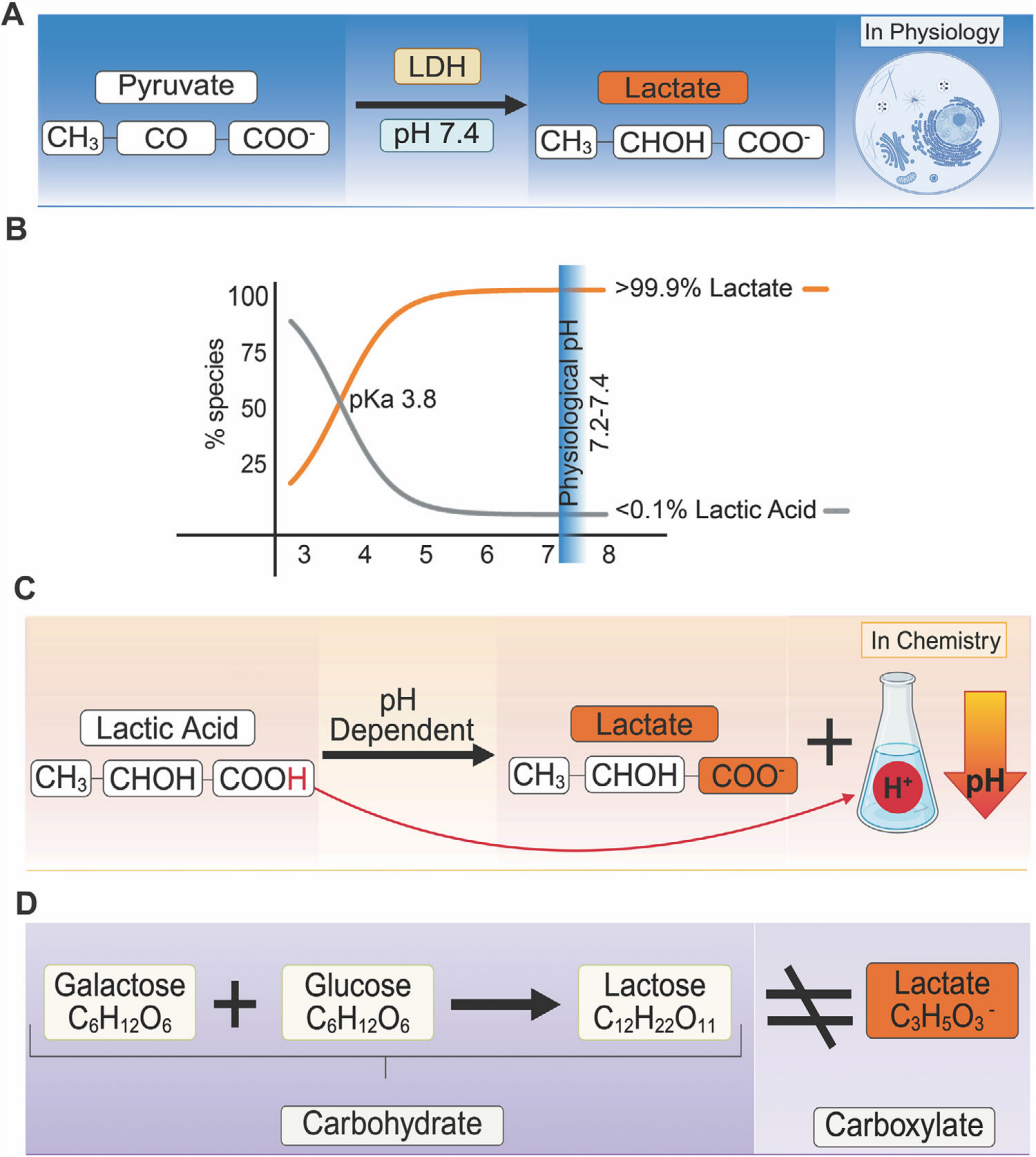
Lactate at Physiological pH: A Metabolite, not an Acid or Sugar. (A) Glycolysis terminates in lactate via the lactate dehydrogenase reaction, not in lactic acid production. The source of lactate in mammalian cells is pyruvate and not lactic acid. (B) Lactate and lactic acid exist as a conjugate base‐acid pair with a pKa of approximately 3.8. At physiological pH (7.2–7.4), more than 99.9% of this species exists in the dissociated lactate form, making accumulation of lactic acid biologically implausible. (C) Lactic acid can dissociate into lactate and a proton, resulting in the acidification of the media. However, this occurs outside physiological conditions. (D) Lactose, a disaccharide composed of glucose and galactose, is chemically distinct and should not be conflated with lactate. Imprecise terminology has contributed substantially to misconceptions surrounding lactate biology.

Mammalian cells do not produce lactic acid. There is no step in human metabolism that produces lactic acid as a product. Glycolysis terminates in lactate via the lactate dehydrogenase (LDH) reaction, not in lactic acid [2, 5]. The term “lactic acid” refers only to the protonated form of lactate, which exists only as a negligible fraction at physiological pH (7.2–7.4), the normal intracellular pH range required for cellular function. At pH 7.2–7.4, with a pKa of approximately 3.8, more than 99.9% of lactate exists in the dissociated form (Figure 1). The pKa represents the pH at which protonated and deprotonated forms are equal, as predicted by the Henderson–Hasselbalch relationship [2, 6]. Accordingly, the term “lactic acid” is largely a historical artifact rather than a physiologically meaningful species under most biological conditions. As a result, lactic acid cannot accumulate as a discrete biological entity in vivo. Importantly, pathological elevations of D‐lactate arise from distinct microbial or gastrointestinal processes and should not be conflated with physiological L‐lactate metabolism in mammalian cells [7].

Confusion between lactate and lactic acid often arises from a lack of awareness of lactate's true biochemical origin. In mammalian cells, lactate is generated from pyruvate through LDH activity, not from lactic acid. Recognizing pyruvate as the source of lactate and that lactic acid does not exist in appreciable amounts at physiological pH is essential to resolving this common misconception [2].

Lactose, by contrast, is a disaccharide composed of glucose and galactose and is chemically unrelated to lactate. Although lactose metabolism can ultimately supply carbon to lactate through glycolysis, the molecules are neither structurally nor functionally equivalent [8]. Failure to distinguish among lactate, lactic acid, and lactose underlies many downstream misconceptions regarding acidosis, fatigue, and pathological lactate accumulation (Figure 1).

### 2.2 Myth 2: Lactate Is Produced Only Under Anaerobic Conditions

#### 2.2.1 Truth: Lactate Is Produced Under All Oxygen Conditions

The association between lactate production and oxygen deficiency originates from early studies of fermentation and ischemia, in which lactate accumulation coincided with low oxygen availability. These observations fostered the enduring belief that lactate is a hallmark of anaerobic metabolism.

In mammalian cells, however, lactate is continuously produced under fully aerobic conditions. Lactate formation reflects the rate of glycolysis and the need to regenerate NAD^+^ to sustain glycolytic flux, rather than oxygen availability alone [1, 3, 9, 10]. The LDH reaction operates near equilibrium and is governed primarily by substrate concentrations and the cytosolic NADH to NAD^+^ ratio.

In cancer, the Warburg effect further illustrates that lactate production in the presence of oxygen does not imply defective respiration; rather, it may reflect a metabolic program that supports anabolic flux, redox balance, or saturation of mitochondrial oxidative capacity. Whether aerobic lactate production in tumors primarily reflects anabolic requirements, redox balancing, thermodynamic constraints, or saturation of mitochondrial oxidative capacity remains an active area of investigation.

**FIGURE 2:**
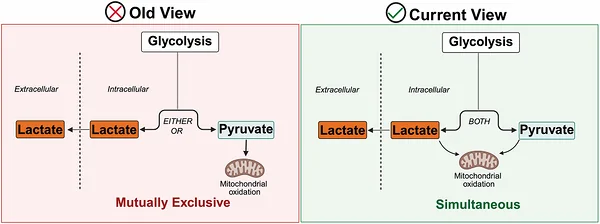
Lactate production and mitochondrial respiration coexist. The traditional view presents pyruvate metabolism as a false dichotomy in which pyruvate is directed either toward lactate production and export or mitochondrial oxidation. This interpretation implies that lactate formation is mutually exclusive with mitochondrial respiration. In contrast, the current view recognizes that pyruvate can simultaneously support lactate production and mitochondrial oxidation. Therefore, elevated lactate production does not necessarily indicate impaired mitochondrial respiration; rather, high lactate output can coexist with active mitochondrial metabolism and should be interpreted in the context of metabolic flux, redox balance, and energetic demand.

Importantly, lactate accumulation does not imply mitochondrial failure. In many contexts, lactate is simultaneously produced and oxidized, reflecting high rates of metabolic flux rather than impaired respiration (Figure 2). This dynamic exchange is consistent with lactate shuttle models, in which lactate serves as a mobile carrier of carbon and reducing equivalents between and within tissues. Elevated lactate commonly occurs in cells with intact and highly active mitochondria operating under high metabolic demand, including proliferating cells, contracting skeletal muscle, and many tumors [3, 10, 11]. When glycolytic flux exceeds the immediate capacity of mitochondrial oxidation or pyruvate utilization, lactate production increases even in the presence of abundant oxygen. Interpreting lactate as a proxy for anaerobiosis therefore obscures its role as a marker of metabolic flux. Although lactate can rise during relative oxygen limitation and may serve as a marker of increased glycolytic reliance in some settings, it is neither specific for anaerobic metabolism nor evidence of mitochondrial failure.

### 2.3 Myth 3: Lactate Is a Metabolic Dead End

#### 2.3.1 Truth: Lactate Is a Dynamic Metabolic Fuel and Not an End Product

Lactate has long been portrayed as the terminal product of glycolysis; a metabolic cul‐de‐sac reached when glucose cannot be fully oxidized. This view framed lactate as metabolic waste rather than as a usable fuel.

In contrast, lactate is a major circulating fuel that is actively exchanged and oxidized by multiple tissues, as demonstrated by arteriovenous balance studies during human exercise [9]. In healthy individuals, circulating lactate is present at low millimolar concentrations even at rest [2, 3]. These basal levels reflect continuous production and utilization rather than metabolic dysfunction. Importantly, relatively stable lactate concentrations can coexist with high rates of lactate turnover and interorgan exchange, further emphasizing that concentration alone does not necessarily reflect metabolic flux.

Isotope tracing studies have fundamentally reshaped this perspective. Quantitative in vivo flux analyses demonstrate that glucose‐derived carbon enters the tricarboxylic acid cycle, in many tissues and physiological contexts, often via circulating lactate rather than direct glucose oxidation, establishing lactate as a central exchange metabolite in carbohydrate metabolism [12, 13]. Consistent with this, arteriovenous metabolomics in humans reveals substantial cardiac lactate uptake under both resting and stressed conditions [14]. Rather than a dead end, lactate serves as a distributed carrier of carbon and reducing equivalents that links glycolysis in one tissue to oxidative metabolism in another.

### 2.4 Myth 4: Lactate Causes Acidosis, Muscle Fatigue, or Pain

#### 2.4.1 Truth: Lactate Generation Does Not Increase Acidosis or Cause Pain

Because lactate levels often rise during intense exercise or illness alongside acidosis, fatigue, and pain, lactate has historically been assumed to be their direct cause. This assumption is incorrect.

**FIGURE 3:**
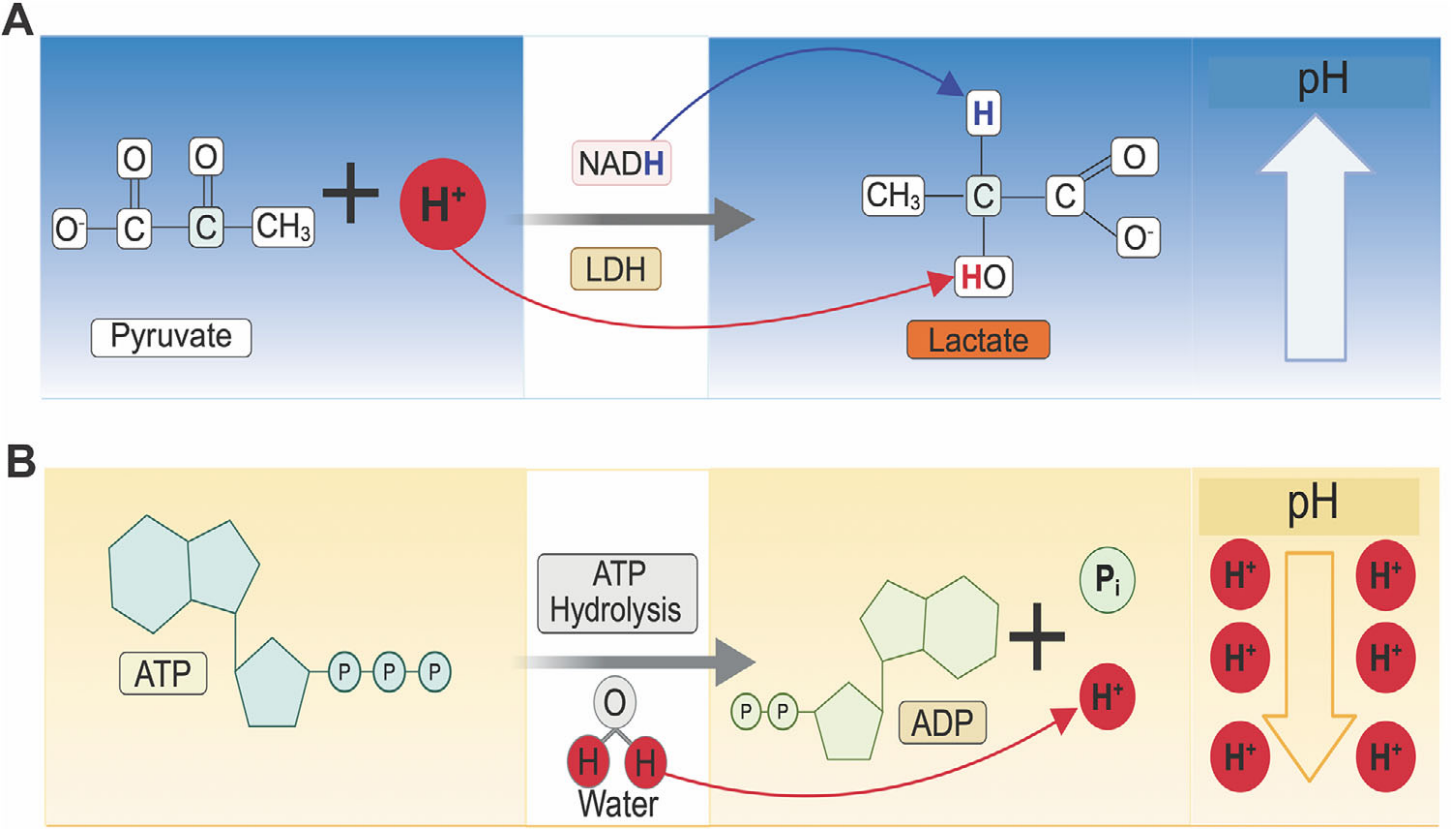
Proton generation and lactate production are mechanistically distinct. (A) The lactate dehydrogenase reaction regenerates NAD^+^ to sustain glycolytic flux and does not generate protons. (B) Proton generation during metabolic stress arises primarily from ATP hydrolysis associated with energy consumption, not from lactate production. Extracellular acidification therefore reflects integrated proton flux from multiple metabolic processes and should not be equated with lactate production. Conflating lactate accumulation with acidosis represents a mechanistic error that obscures the true sources of pH change.

Lactate itself does not generate protons and therefore does not directly lower pH under physiological conditions. In fact, when considered at the level of net proton balance, lactate production does not contribute to acidosis. Net proton production arises primarily from ATP hydrolysis during energy consumption, not from lactate formation [2, 5]. Lactate production can instead help buffer cytosolic redox state by regenerating NAD^+^, thereby supporting continued ATP production through glycolysis (Figure 3). In fact, the LDH reaction consumes a proton during the conversion of pyruvate to lactate, further dissociating lactate production from acidosis.

**FIGURE 4:**
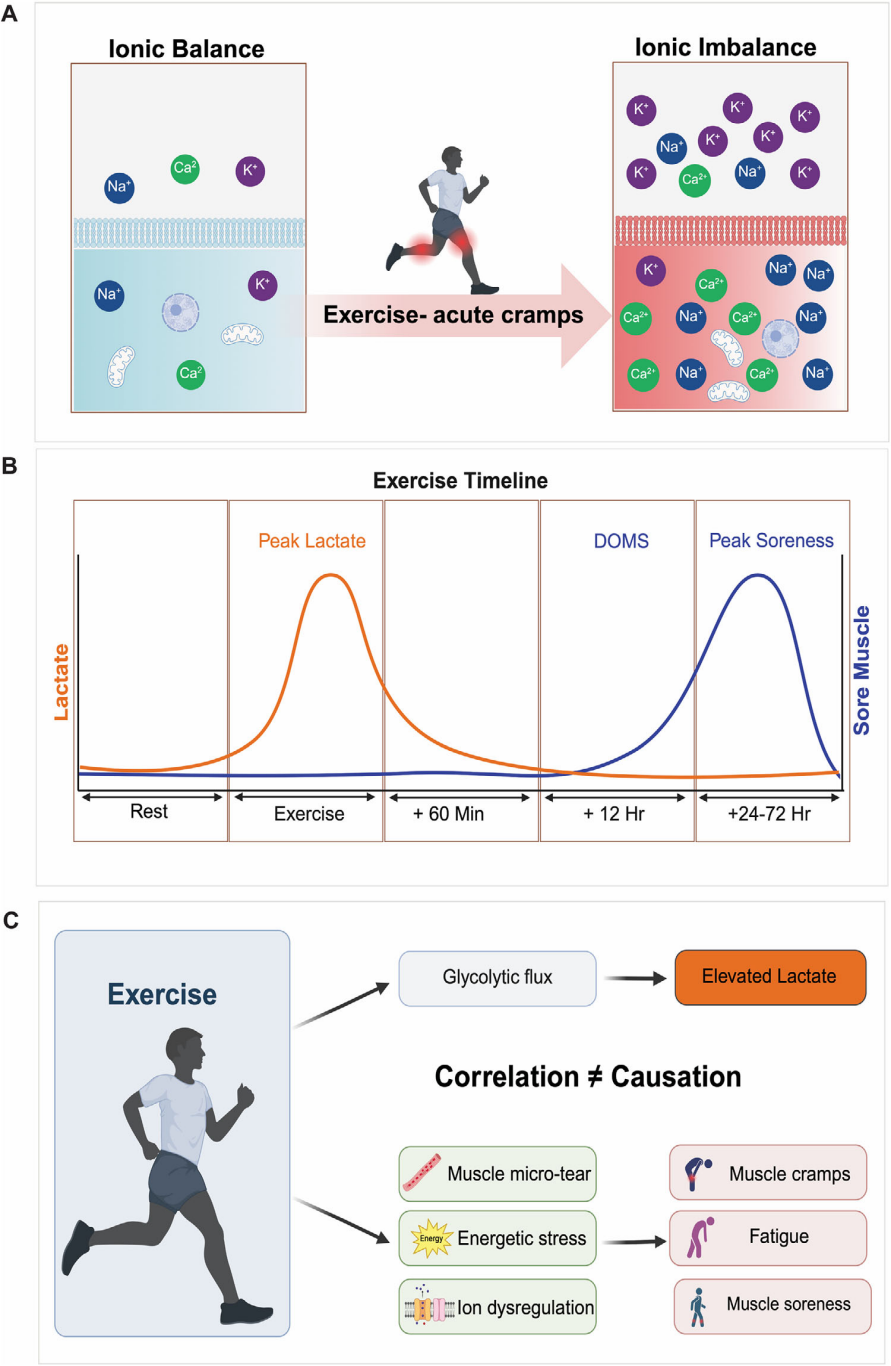
Temporal dissociation between lactate accumulation and muscle soreness following exercise. (A) Under resting conditions, Na^+^, K^+^, and Ca^2^^+^ gradients across the muscle cell membrane are tightly regulated to support normal membrane excitability and contraction. During intense or prolonged exercise, repeated muscle activation and metabolic stress can disturb local ion distribution and membrane excitability, contributing to involuntary and painful muscle contractions. The illustration is schematic and does not indicate absolute ion concentrations. (B) Delayed‐onset muscle soreness (DOMS) is not caused by lactate. During exercise, lactate levels rise rapidly in proportion to glycolytic flux, while muscle soreness remains minimal. About an hour after exercise, lactate has largely returned toward baseline, with little change in soreness. Twelve hours after exercise, lactate remains low, whereas DOMS initiates. Twenty‐four hours after exercise, lactate remains at baseline, and muscle soreness still peaks and resolves by 72 h. This figure highlights that lactate accumulation is transient and temporally uncoupled from the development of postexercise muscle soreness. (C) Exercise increases glycolytic flux and consequently elevates lactate production. However, the simultaneous occurrence of elevated lactate with muscle cramps, fatigue, and soreness does not establish a causal relationship. These symptoms are more directly associated with overlapping exercise‐induced processes, including ion dysregulation, increased energetic demand, and muscle microdamage.

Experimental and physiological evidence demonstrates that lactate does not impair force production and can delay fatigue. Muscle pain, cramping, and contractile dysfunction instead arise from ionic disturbances, altered calcium handling, and impaired excitation‐contraction coupling [15]. Early morphological studies of delayed‐onset muscle soreness demonstrated structural disruption of muscle fibers following exercise, providing direct evidence that soreness reflects mechanical damage rather than metabolite accumulation such as lactate [16]. The rapid clearance of lactate following exercise, well before the onset of delayed‐onset muscle soreness, further argues against a causal role for lactate. Resolving these misconceptions requires not only mechanistic clarity but also careful consideration of how lactate is measured and interpreted experimentally. Subsequent studies using imaging, histological, and biochemical approaches have confirmed that delayed‐onset muscle soreness (DOMS) is driven by structural muscle damage, neural muscle damage, and inflammation rather than metabolite accumulation [17, 18, 19]. Figure 4 emphasizes that the delayed timing of DOMS is incompatible with a causal role for lactate accumulation and instead aligns with the time course of mechanical damage and inflammatory repair.

### 2.5 Myth 5: Elevated Lactate Is Inherently Harmful or Synonymous With Hypoxia

#### 2.5.1 Truth: Elevated Lactate Reflects Metabolic Context Rather Than Oxygen Availability Alone

Historically, the association between lactate and critical illness can be traced to early clinical observations of elevated blood lactate in shock, long before mechanistic understanding of metabolism or perfusion was available [20].

In clinical settings, elevated lactate is often interpreted as definitive evidence of tissue hypoxia or metabolic failure. In reality, hyperlactatemia reflects a range of physiological and pathological processes, including adrenergic stimulation, altered clearance, mitochondrial reprogramming, and changes in tissue perfusion [21, 22, 23].

Importantly, lactate is a valuable prognostic biomarker and has saved lives in critical care. The key distinction lies between lactate as a signal of disease severity and lactate as a direct causal agent of pathology. Elevated lactate is not intrinsically toxic, nor is it specific for hypoxia. Confusing these roles has contributed to persistent misinterpretations of lactate biology and to inappropriate therapeutic assumptions [24]. Importantly, recognizing lactate as a correlational biomarker rather than a causal toxin does not diminish its prognostic utility, but rather clarifies how it should be interpreted alongside perfusion, clearance, and metabolic context.

### 2.6 Myth 6: Lactate Is Metabolically Passive

#### 2.6.1 Truth: Lactate Is a Metabolically Active Molecule With Several Functions

Lactate was long viewed as a metabolically inert end product whose relevance ended with its oxidation. This view has been substantially revised. Beyond serving as a fuel, lactate participates in regulatory processes that link metabolic state to cellular behavior. One such mechanism is protein lactylation, a post‐translational modification in which lactate‐derived carbon is covalently attached to lysine residues. Histone lactylation provides a direct connection between cellular metabolism and gene expression [25]. Recent work has further demonstrated enzymatic control of this process by identifying the histone acetyltransferase HBO1 as a histone lactyltransferase [26]. Furthermore, lactylation links cellular metabolism to post‐transcriptional RNA regulation by RNA‐modifying enzymes such as nudix hydrolase‐21 (NUDT21) [27].

Lactylation is not restricted to histones. Nonhistone protein lactylation can exert direct functional effects on cellular physiology. For example, lactylation of the DNA repair protein NBS1 alters DNA damage responses and contributes to chemotherapeutic resistance [28]. In addition, lactate can serve as a precursor for bioactive metabolites. Exercise induces the production of N‐lactoyl‐phenylalanine, a circulating metabolite associated with reduced feeding and adiposity [29]. Together, these findings support the emerging view that lactate can participate in metabolic regulation in specific contexts, rather than functioning solely as a passive metabolic intermediate.

This evolving understanding reframes lactate not as a pathological by‐product, but as an important integrator of metabolic flux, redox state, and cellular regulation. These advances further motivate a re‐examination of how lactate is measured and interpreted both experimentally and clinically. Collectively, these misconceptions arise not from lactate biology itself, but from how lactate is measured, contextualized, and interpreted. These regulatory roles are highly context‐dependent and should not be interpreted as universal effects of lactate across all cell types or conditions.

## 3 Discussion

### 3.1 Lactate as a Central Integrator of Metabolic State

Correcting misconceptions about lactate does more than resolve semantic confusion; it reshapes how metabolism is interpreted experimentally and clinically. Many of these misconceptions persisted not because the underlying biology was simple, but because earlier experimental approaches lacked the spatial resolution, temporal resolution, and physiological context required to capture lactate dynamics accurately. These misconceptions are further reinforced by limitations of experimental systems and measurement strategies used to study it.

### 3.2 How We Measure Lactate Shapes What We Conclude

Advances in methodology have been central to reshaping our understanding of lactate biology. Importantly, each experimental approach captures a different aspect of lactate metabolism, and no single method provides a complete picture. Failure to distinguish among concentration, pool size, and metabolic flux has contributed substantially to persistent misconceptions. Thus, lactate concentration alone is insufficient to infer metabolic flux or directionality.

Early studies using radiolabeled substrates, including ^14^C‐ and tritium‐labeled glucose and lactate, were instrumental in establishing foundational concepts such as the Cori cycle and interorgan lactate exchange. These approaches demonstrated that lactate is actively produced and utilized across tissues, including the liver and heart. However, such methods require terminal sampling or invasive biopsies, limiting spatial resolution while capturing population‐averaged behavior. As a result, they were poorly suited to resolving rapid or compartment‐specific metabolic dynamics.

The development of stable isotope tracing combined with mass spectrometry and NMR represented a major advance, enabling quantitative analysis of metabolic fluxes in vivo. These approaches revealed that glucose‐derived carbon frequently enters the tricarboxylic acid cycle via circulating lactate, fundamentally revising the view of lactate as a metabolic endpoint. However, these methods typically rely on assumptions of isotopic and metabolic steady state, which are difficult to satisfy in dynamic physiological conditions such as exercise or ischemia‐reperfusion. Importantly, conventional isotope tracing approaches cannot readily resolve simultaneous bidirectional fluxes, a key feature of lactate metabolism in vivo. Additional NMR‐based approaches such as saturation transfer can, in select settings, help establish the chemical origin and exchange relationships of metabolites, further complementing tracer‐based and sensor‐based methods.

More recent approaches have begun to address these limitations. Hyperpolarized ^13^C magnetic resonance spectroscopy represents a major conceptual advance by enabling real‐time, in vivo measurement of metabolic flux, thereby enabling direct assessment of metabolic exchange flux rather than static metabolite pool size, a key limitation of earlier methods [30]. This technique allows direct observation of pyruvate‐to‐lactate isotopic exchange through lactate dehydrogenase, providing a dynamic readout of flux rather than static metabolite levels. These studies have demonstrated that lactate production and utilization can occur concurrently within living tissues, challenging the notion of lactate as a unidirectional end product and reinforcing the concept of lactate as a dynamic intermediary within distributed metabolic networks.

Genetically encoded lactate sensors offer complementary strengths by providing high spatial and temporal resolution within cells. These tools have revealed substantial heterogeneity in lactate distribution across subcellular compartments. However, such sensors primarily report metabolite concentration rather than flux and are generally limited to experimentally accessible tissues or model systems.

In clinical practice, lactate is typically measured using enzymatic assays for total L‐lactate and remains vulnerable to pre‐analytical variables such as sample handling, delayed processing, hemolysis, and differences in point‐of‐care device performance, particularly at the extremes of the physiological range.

Taken together, these approaches highlight a central principle: different methods measure fundamentally different aspects of lactate biology. Misinterpretation frequently arises when concentration is equated with flux, when systemic measurements are assumed to reflect local metabolism, or when temporally dynamic processes are interpreted from static measurements. Recognizing these distinctions is essential for correctly interpreting lactate in both experimental and clinical contexts.

### 3.3 Experimental Context and Model Systems

Excluding lactate from in vitro culture media creates an artificial metabolic environment that limits lactate uptake, oxidation, and redox buffering by eliminating extracellular lactate as a physiological substrate and redox partner [31, 32]. In such settings, cells are forced into nonphysiological metabolic states, biasing interpretation toward lactate as an endpoint rather than an active intermediary.

A related source of confusion arises from the interpretation of extracellular acidification as a direct measure of lactate production. Changes in extracellular pH reflect net proton flux, which arises primarily from ATP hydrolysis during energy consumption, not from lactate flux. Conflating these readouts therefore mistakes acidity for lactate generation and perpetuates the erroneous notion that lactic acid is being produced [2, 5]. Although extracellular acidification measurements can serve as general indicators of metabolic activity, they should not be equated with lactate production.

These limitations foster broader interpretive errors in lactate biology. Lactate frequently accompanies metabolic stress, but this association does not imply that lactate is the source of the underlying dysfunction. A useful analogy is that of firefighters and fires. Firefighters are reliably present at fires, but their presence does not imply causation. Similarly, lactate frequently accompanies metabolic stress without necessarily serving as the underlying driver of pathology. Relying on indirect readouts or simplified experimental systems increases the risk of misattributing association to mechanism.

Interpretation of lactate measurements is further complicated by how lactate is typically assessed. Most experimental and clinical measurements rely on blood lactate, which represents a composite systemic signal integrating production, utilization, and clearance across multiple organs and tissues, often at single time points [21, 23]. This global readout can obscure substantial heterogeneity in intracellular and compartment‐specific lactate concentrations and provides limited insight into local metabolic states.

Recent advances in genetically encoded lactate sensors and isotope tracing have revealed pronounced spatial heterogeneity in lactate distribution. Lactate levels can differ markedly between cytosol, mitochondria, and extracellular space, even within the same cell [33, 34]. These findings underscore that lactate concentration is not uniform, and that local lactate availability may diverge substantially from circulating measurements. Consequently, elevated blood lactate does not necessarily indicate elevated intracellular lactate in all tissues, nor does it predict impaired mitochondrial oxidation. As tools for compartment‐resolved metabolic analysis continue to improve, aligning lactate measurements with specific biological questions will be essential. Treating lactate as a single global proxy for metabolic state risks obscuring meaningful spatial and contextual differences.

From an experimental perspective, distinguishing correlation from causation in lactate biology requires approaches that directly perturb and trace metabolic pathways. This is particularly important for lactate, where elevated levels often reflect adaptive metabolic responses rather than direct pathological drivers. These include isotope tracing to quantify flux, genetic, or pharmacological manipulation of key enzymes and transporters such as LDH and monocarboxylate transporters, and compartment‐resolved measurements to capture spatial dynamics. Integrating these approaches allows lactate to be interpreted within the broader context of metabolic network behavior rather than as an isolated readout. These experimental limitations are particularly relevant when interpreting mitochondrial lactate metabolism, where compartmentalization and flux directionality remain difficult to resolve.

### 3.4 Lactate and Mitochondrial Metabolism

The concept of mitochondrial lactate metabolism is not new and has evolved alongside improvements in metabolic measurement and subcellular resolution. Early physiological and biochemical studies provided evidence for intracellular lactate oxidation and reported lactate dehydrogenase activity and monocarboxylate transporter immunoreactivity associated with mitochondrial fractions in select tissues [1, 3, 35]. Although these studies predated modern imaging approaches and did not resolve transport mechanisms or subcellular localization with precision, they established the foundational idea that lactate can, under specific conditions, participate directly in mitochondrial metabolism.

Recent advances have provided critical spatial and functional clarity. Using the ultrasensitive FiLa biosensor, lactate was shown to be highly enriched within mammalian mitochondria, revealing pronounced subcellular lactate gradients in living cells and in vivo [34]. These observations demonstrate that mitochondria are not isolated from lactate but instead exist within a lactate‐rich microenvironment and can contain substantial amounts of lactate under normal physiological conditions.

Importantly, mitochondrial lactate enrichment does not imply uniform lactate utilization across all cell types or tissues. Rather, multiple independent studies demonstrate that, in specific physiological and pathological contexts, mitochondrial lactate is not merely present but is actively oxidized and used as a fuel and redox substrate. In these settings, lactate contributes both carbon and reducing equivalents to oxidative metabolism in skeletal muscle, immune cells, and heart [11, 12, 13, 14], consistent with earlier lactate shuttle models [3]. These findings indicate that lactate oxidation can coexist with, and complement, other mitochondrial substrates depending on tissue identity, metabolic demand, and physiological context.

Beyond acute energy production, lactate availability can also influence longer‐term mitochondrial programs. During trained immunity, lactate has been shown to support mitochondrial metabolism and epigenetic remodeling, illustrating how lactate‐dependent metabolic states can shape sustained cellular responses beyond immediate energetic needs [36]. This example highlights that lactate's mitochondrial roles are not limited to short‐term fuel provision.

Notably, in a model of severe early‐onset mitochondrial myopathy, in vivo tracing and metabolic analyses indicate that diseased muscle becomes unusually dependent on lactate import and utilization [33]. In this context, lactate functions as a critical compensatory substrate when conventional oxidative metabolism is compromised. Taken together, differences in tissue type, metabolic demand, and experimental context likely account for apparent inconsistencies in earlier literature, underscoring the context‐dependent nature of mitochondrial lactate metabolism.

### 3.5 Lactate Beyond Fuel: Signaling and Regulation

Beyond its role as a metabolic fuel, lactate can influence cellular regulation through downstream metabolites and post‐translational modifications that link metabolic state to gene expression and protein function. One such mechanism is lysine lactylation, a post‐translational modification in which lactate‐derived carbon is covalently attached to lysine residues. Lactate availability contributes to lactylation through lactoyl‐CoA and lactoyl‐glutathione‐dependent pathways, providing a direct biochemical link between cellular metabolism and transcriptional control [25, 27, 37, 38].

Lactylation is not restricted to histones. Recent work has demonstrated that nonhistone protein lactylation can exert direct functional effects on cellular physiology. For example, lactylation of the DNA repair protein NBS1 alters DNA damage responses and contributes to chemotherapeutic resistance, establishing lactylation as a regulatory post‐translational modification capable of modulating protein function and cellular phenotypes [28].

In addition to protein modification, lactate can serve as a precursor for bioactive metabolites that act at the organismal level. Exercise induces the production of N‐lactoyl‐phenylalanine (Lac‐Phe), a circulating metabolite derived from lactate that is associated with reduced feeding and adiposity, illustrating how lactate‐dependent metabolic flux can be translated into systemic physiological responses [29].

Beyond these indirect regulatory routes, lactate can also directly regulate protein function through specific metabolite‐protein interactions. Recent work demonstrated that lactate binds within a zinc‐containing active site of the SUMO protease SENP1, forming a lactate‐zinc complex that inhibits SENP1 activity and alters mitotic progression [39]. This interaction does not imply nonspecific zinc chelation by lactate but rather illustrates how metabolites can exert precise and context‐dependent regulatory effects on protein activity. Together, these findings suggest that lactate biology extends well beyond canonical energy metabolism and remains incompletely understood.

### 3.6 Still at the Tip of the Iceberg

Viewed collectively, lactate occupies a central position at the intersection of fuel distribution, redox homeostasis, mitochondrial function, and metabolic regulation. That such diverse roles continue to emerge from a metabolite long dismissed as waste underscores how incomplete prevailing models of metabolism remain. A detailed mechanistic and historical framework for lactate routing, compartmentalization, and molecular diversification is beyond the scope of this review and will be addressed separately. Many of the myths addressed in this review persist precisely because earlier experimental approaches lacked the spatial resolution and physiological context required to capture lactate dynamics accurately. As experimental tools continue to advance, it is likely that additional context‐specific roles for lactate will be uncovered, further expanding its biological and clinical relevance across physiology and disease.

### 3.7 Why Misconceptions Persist

Despite substantial advances in metabolic research, misconceptions about lactate remain remarkably durable. This persistence reflects not only gaps in experimental resolution but also fundamental features of how scientific knowledge is constructed and transmitted.

One contributing factor is the human tendency to favor simple causal narratives over probabilistic or systems‐level reasoning. Lactate frequently rises in conditions of physiological stress or disease, and this association invites intuitive but often incorrect conclusions about causality. Interpreting lactate as the driver of pathology, rather than as a marker of underlying metabolic processes, reflects a broader cognitive bias toward narrative explanations over conditional reasoning.

A second factor is the mismatch between biological complexity and measurement resolution. Lactate dynamics span multiple spatial scales—from subcellular compartments to whole‐organism circulation—and evolve across timescales ranging from seconds to days. In clinical settings, circulating lactate reflects integrated systemic metabolism and may not directly report tissue‐specific metabolic states. When measurement bandwidth is narrower than biological complexity, simplified interpretations tend to fill the gap.

Finally, educational frameworks play a role. Simplified models of metabolism are often introduced for pedagogical clarity and can persist long after they have been revised in the primary literature. Concepts such as lactate as a marker of anaerobic metabolism remain deeply embedded within textbooks, physiology curricula, and clinical heuristics, reinforcing outdated interpretations across generations of scientists and clinicians.

Correcting these misconceptions will require not only improved experimental tools, but also changes in how metabolism is conceptualized, taught, and communicated across training environments.

## 4 Conclusion

Lactate has long been burdened by misconceptions that conflate correlation with causation. Rather than serving as a toxic by‐product or a marker of anaerobic failure, lactate is a central intermediary in energy metabolism, redox homeostasis, and inter‐organ metabolic communication. Lactate is not the problem; it is a key part of the solution.

## Author Contributions

A.A.C. conceived the review and led manuscript development and revision. B.B. performed the literature review, generated all figures, and prepared the initial manuscript draft. All authors contributed to manuscript editing, discussion, and approved the final version of the manuscript.

## Funding

This work was supported by a National Institutes of Health Pathway to Independence Award (R00HL168312) to A.A.C. B.B. is supported by the Rutgers INSPIRE (IRACDA New Jersey/New York for Science Partnerships in Research and Education) Postdoctoral Fellowship.

## Conflicts of Interest

The authors declare no conflicts of interest.

## Data Availability

No new data were generated or analyzed in this study.

## References

[1] L. B. Gladden , “Lactate Metabolism: A New Paradigm for the Third Millennium,” The Journal of Physiology 558, no. Pt 1 (2004): 5–30, 10.1113/jphysiol.2003.058701. 15131240 PMC1664920

[2] R. A. Robergs , et al., “Lactate, Not Lactic Acid, Is Produced by Cellular Cytosolic Energy Catabolism,” Physiology (Bethesda) 33, no. 1 (2018): 10–12. 29212886 10.1152/physiol.00033.2017

[3] G. A. Brooks , “Cell–cell and Intracellular Lactate Shuttles,” The Journal of Physiology 587, no. Pt 23 (2009): 5591–5600, 10.1113/jphysiol.2009.178350. 19805739 PMC2805372

[4] H. Schneider , J. Danko , R. Huch , and A. Huch , “Homeostasis of Fetal Lactate Metabolism in Late Pregnancy and the Changes During Labor and Delivery,” European Journal of Obstetrics & Gynecology and Reproductive Biology 17, no. 2‐3 (1984): 183–192, 10.1016/0028-2243(84)90142-4. 6376200

[5] R. A. Robergs , F. Ghiasvand , and D. Parker , “Biochemistry of Exercise‐induced Metabolic Acidosis,” American Journal of Physiology‐Regulatory, Integrative and Comparative Physiology 287, no. 3 (2004): R502–R516, 10.1152/ajpregu.00114.2004. 15308499

[6] D. H. Williamson , P. Lund , and H. A. Krebs , “The Redox state of Free Nicotinamide‐adenine Dinucleotide in the Cytoplasm and Mitochondria of Rat Liver,” Biochemical Journal 103, no. 2 (1967): 514–527, 10.1042/bj1030514. 4291787 PMC1270436

[7] J. B. Ewaschuk , J. M. Naylor , and G. A. Zello , “D‐lactate in Human and Ruminant Metabolism,” The Journal of Nutrition 135, no. 7 (2005): 1619–1625, 10.1093/jn/135.7.1619. 15987839

[8] D. L. Nelson and M. M. Cox , “Lehninger Principles of Biochemistry,” Seventh edition. ed. 2017 W.H. Freeman.

[9] G. Van Hall , et al., “Leg and Arm Lactate and Substrate Kinetics During Exercise,” American Journal of Physiology Endocrinology and Metabolism 284, no. 1 (2003): E193–205. 12388120 10.1152/ajpendo.00273.2002

[10] G. A. Brooks , “The Science and Translation of Lactate Shuttle Theory,” Cell Metabolism 27, no. 4 (2018): 757–785, 10.1016/j.cmet.2018.03.008. 29617642

[11] B. Faubert , K. Y. Li , L. Cai , et al., “Lactate Metabolism in Human Lung Tumors,” Cell 171, no. 2 (2017): 358–371.e9, 10.1016/j.cell.2017.09.019, e9. 28985563 PMC5684706

[12] S. Hui , J. M. Ghergurovich , R. J. Morscher , et al., “Glucose Feeds the TCA Cycle via Circulating Lactate,” Nature 551, no. 7678 (2017): 115–118, 10.1038/nature24057. 29045397 PMC5898814

[13] S. Hui , A. J. Cowan , X. Zeng , et al., “Quantitative Fluxomics of Circulating Metabolites,” Cell Metabolism 32, no. 4 (2020): 676–688.e4, 10.1016/j.cmet.2020.07.013. 32791100 PMC7544659

[14] D. Murashige , C. Jang , M. Neinast , et al., “Comprehensive Quantification of Fuel Use by the Failing and Nonfailing human Heart,” Science 370, no. 6514 (2020): 364–368, 10.1126/science.abc8861. 33060364 PMC7871704

[15] D. G. Allen , G. D. Lamb , and H. Westerblad , “Skeletal Muscle Fatigue: Cellular Mechanisms,” Physiological Reviews 88, no. 1 (2008): 287–332, 10.1152/physrev.00015.2007. 18195089

[16] J. Fridén , M. Sjöström , and B. Ekblom , “A Morphological Study of Delayed Muscle Soreness,” Experientia 37, no. 5 (1981): 506–507, 10.1007/BF01986165. 7250326

[17] U. Proske and D. L. Morgan , “Muscle Damage From Eccentric Exercise: Mechanism, Mechanical Signs, Adaptation and Clinical Applications,” The Journal of Physiology 537, no. Pt 2 (2001): 333–345, 10.1111/j.1469-7793.2001.00333.x. 11731568 PMC2278966

[18] K. Mizumura and T. Taguchi , “Delayed Onset Muscle Soreness: Involvement of Neurotrophic Factors,” The Journal of Physiological Sciences 66, no. 1 (2016): 43–52, 10.1007/s12576-015-0397-0. 26467448 PMC10716961

[19] B. Sonkodi , I. Berkes , and E. Koltai , “Have We Looked in the Wrong Direction for More than 100 Years? Delayed Onset Muscle Soreness Is,” Fact, Neural Microdamage Rather Than Muscle Damage Antioxidants (Basel) 9, no. 3 (2020): 212. 32150878 10.3390/antiox9030212 PMC7139782

[20] E. J. O. Kompanje , T. C. Jansen , B. Van Der Hoven , and J. Bakker , “The First Demonstration of Lactic Acid in Human Blood in Shock by Johann Joseph Scherer (1814–1869) in January 1843,” Intensive Care Medicine 33, no. 11 (2007): 1967–1971, 10.1007/s00134-007-0788-7. 17661014 PMC2040486

[21] L. W. Andersen , J. Mackenhauer , J. C. Roberts , K. M. Berg , M. N. Cocchi , and M. W. Donnino , “Etiology and Therapeutic Approach to Elevated Lactate Levels,” Mayo Clinic Proceedings 88, no. 10 (2013): 1127–1140, 10.1016/j.mayocp.2013.06.012. 24079682 PMC3975915

[22] B. Suetrong and K. R. Walley , “Lactic Acidosis in Sepsis: It's Not All Anaerobic,” Chest 149, no. 1 (2016): 252–261, 10.1378/chest.15-1703. 26378980

[23] J. A. Kraut and N. E. Madias , “Lactic Acidosis,” New England Journal of Medicine 371, no. 24 (2014): 2309–2319, 10.1056/NEJMra1309483. 25494270

[24] W. D. Lee , D. R. Weilandt , L. Liang , et al., “Lactate Homeostasis Is Maintained Through Regulation of Glycolysis and Lipolysis,” Cell Metabolism 37, no. 3 (2025): 758–771.e8, 10.1016/j.cmet.2024.12.009, e8. 39889702 PMC11926601

[25] D. Zhang , Z. Tang , H. Huang , et al., “Metabolic Regulation of Gene Expression by Histone Lactylation,” Nature 574, no. 7779 (2019): 575–580, 10.1038/s41586-019-1678-1. 31645732 PMC6818755

[26] Z. Niu , C. Chen , S. Wang , et al., “HBO1 catalyzes Lysine Lactylation and Mediates Histone H3K9la to Regulate Gene Transcription,” Nature Communications 15, no. 1 (2024): 3561, 10.1038/s41467-024-47900-6. PMC11053077 38670996

[27] J. Lin , Y. Yin , J. Cao , et al., “NUDT21 lactylation Reprograms Alternative Polyadenylation to Promote Cuproptosis Resistance,” Cell Discovery 11, no. 1 (2025): 52, 10.1038/s41421-025-00804-1. 40425546 PMC12116747

[28] H. Chen , Y. Li , H. Li , et al., “NBS1 lactylation Is Required for Efficient DNA Repair and Chemotherapy Resistance,” Nature 631, no. 8021 (2024): 663–669, 10.1038/s41586-024-07620-9. 38961290 PMC11254748

[29] V. L. Li , Y. He , K. Contrepois , et al., “An Exercise‐inducible Metabolite That Suppresses Feeding and Obesity,” Nature 606, no. 7915 (2022): 785–790, 10.1038/s41586-022-04828-5. 35705806 PMC9767481

[30] J. H. Ardenkjaer‐Larsen , et al., “Increase in Signal‐to‐noise Ratio of >10,000 Times in Liquid‐state NMR,” PNAS 100, no. 18 (2003): 10158–10163. 12930897 10.1073/pnas.1733835100 PMC193532

[31] J. R. Cantor , M. Abu‐Remaileh , N. Kanarek , et al., “Physiologic Medium Rewires Cellular Metabolism and Reveals Uric Acid as an Endogenous Inhibitor of UMP Synthase,” Cell 169, no. 2 (2017): 258–272.e17, 10.1016/j.cell.2017.03.023. 28388410 PMC5421364

[32] L. B. Sullivan , D. Y. Gui , A. M. Hosios , L. N. Bush , E. Freinkman , and M. G. Vander Heiden , “Supporting Aspartate Biosynthesis Is an Essential Function of Respiration in Proliferating Cells,” Cell 162, no. 3 (2015): 552–563, 10.1016/j.cell.2015.07.017. 26232225 PMC4522278

[33] X. Li , Y. Zhang , L. Xu , et al., “Ultrasensitive Sensors Reveal the Spatiotemporal Landscape of Lactate Metabolism in Physiology and Disease,” Cell Metabolism 35, no. 1 (2023): 200–211.e9, 10.1016/j.cmet.2022.10.002. 36309010 PMC10560847

[34] A. San Martin , et al., “A Genetically Encoded FRET Lactate Sensor and Its Use to Detect the Warburg Effect in Single Cancer Cells,” PLoS ONE 8, no. 2 (2013): 57712. 10.1371/journal.pone.0057712 PMC3582500 23469056

[35] C. Juel and A. P. Halestrap , “Lactate Transport in Skeletal Muscle — Role and Regulation of the Monocarboxylate Transporter,” The Journal of Physiology 517 (1999): 633–642, 10.1111/j.1469-7793.1999.0633s.x. 10358105 PMC2269375

[36] H. Cai , X. Chen , Y. Liu , et al., “Lactate Activates Trained Immunity by Fueling the Tricarboxylic Acid Cycle and Regulating Histone Lactylation,” Nature Communications 16, no. 1 (2025): 3230, 10.1038/s41467-025-58563-2. PMC11971257 40185732

[37] D. O. Gaffney , E. Q. Jennings , C. C. Anderson , et al., “Non‐enzymatic Lysine Lactoylation of Glycolytic Enzymes,” Cell Chemical Biology 27, no. 2 (2020): 206–213.e6, 10.1016/j.chembiol.2019.11.005, e6. 31767537 PMC7395678

[38] R. Liu , X. Ren , Y. E. Park , et al., “Nuclear GTPSCS Functions as a Lactyl‐CoA Synthetase to Promote Histone Lactylation and Gliomagenesis,” Cell Metabolism 37, no. 2 (2025): 377–394.e9, 10.1016/j.cmet.2024.11.005e9. 39642882 PMC11798710

[39] W. Liu , Y. Wang , L. H. M. Bozi , et al., “Lactate Regulates Cell Cycle by Remodelling the Anaphase Promoting Complex,” Nature 616, no. 7958 (2023): 790–797, 10.1038/s41586-023-05939-3. 36921622 PMC12175651

